# Gender-Specific Metabolic Responses of *Crassostrea hongkongensis* to Infection with *Vibrio harveyi* and Lipopolysaccharide

**DOI:** 10.3390/antiox11061178

**Published:** 2022-06-15

**Authors:** Lijuan Ma, Jie Lu, Tuo Yao, Lingtong Ye, Jiangyong Wang

**Affiliations:** 1 Key Laboratory of South China Sea Fishery Resources Exploitation & Utilization, Ministry of Agriculture, South China Sea Fisheries Research Institute, Chinese Academy of Fishery Sciences, Guangzhou 510300, China; 82101202453@caas.cn (L.M.); yaotuo@scsfri.ac.cn (T.Y.); 2 Chinese Academy of Agricultural Sciences, Beijing 100081, China; 3 Key Laboratory of Aquatic Product Processing, Ministry of Agriculture and Rural Affairs, South China Sea Fisheries Research Institute, Chinese Academy of Fishery Sciences, Guangzhou 510300, China; ltye@scsfri.ac.cn; 4 School of Life Science, Huizhou University, Huizhou 516007, China

**Keywords:** *Crassostrea hongkongensis*, metabolomics, hepatopancreas, gender-based difference

## Abstract

Gender differences in the hemocyte immune response of Hong Kong oyster *Crassostrea hongkongensis* to *Vibrio harveyi* and lipopolysaccharide (LPS) infection exist. To determine if a gender difference also exists, we use a ^1^H NMR-based metabolomics method to investigate responses in *C. hongkongensis* hepatopancreas tissues to *V. harveyi* and LPS infection. Both infections induced pronounced gender- and immune-specific metabolic responses in hepatopancreas tissues. Responses are mainly presented in changes in substances involved in energy metabolism (decreased glucose, ATP, and AMP in males and increased ATP and AMP in LPS-infected females), oxidative stress (decreased glutathione in males and decreased tryptophan and phenylalanine and increased choline and proline in LPS-infected females), tricarboxylic acid (TCA) cycle (decreased α-ketoglutarate acid and increased fumarate in LPS-infected males, and decreased fumarate in LPS-infected females), and osmotic regulation (decreased trigonelline and increased taurine in *V. harveyi*-infected males and decreased betaine in *V. harveyi*-infected females). Results suggest that post-spawning-phase male oysters have a more significant energy metabolic response and greater ability to cope with oxidative stress than female oysters. We propose that the impact of oyster gender should be taken into consideration in the aftermath of oyster farming or oyster disease in natural seas.

## 1. Introduction

*Crassostrea hongkongensis* is the most important, commercially valuable cultured oyster species along the South China coast [1,2]. In recent years, the commercial production of this species has been seriously affected by mass mortality during the boreal spring, possibly caused by pathogen infection and environmental stress [3,4]. Therefore, understanding the mechanisms by which oysters respond to different stresses will improve disease control in farmed populations. The genes *ChBeclin-1* [5], *ChUb_L40_* [6], *ChAkt1* [7], *Ch*DFFA [8], and p38 MAPKs [9] play important roles in the immune defense of *C. hongkongensis* against bacterial challenge. The assembled whole genome sequences of *C. hongkongensis* have recently been released [1]. Several immune-, reproduction-, and stress-related genes have been identified [10], providing the resources and opportunity of in-depth studies at the molecular level. Moreover, metabolomics has revealed that exposure to copper can disturb osmotic regulation and energy metabolism [11]. Proteomic methods to analyze differentially expressed proteins in oyster gills exposed to long-term heavy metal pollution have also revealed most stress and immune-reactive proteins, such as heat shock proteins (HSP) and enzymes, to be significantly down-regulated [12].

Development of metabolomic techniques in aquaculture has led to more thorough investigations of shellfish immunology. Physiological and stress responses of bivalves have now been characterized using a variety of metabolomics platforms [13]. Nuclear magnetic resonance (NMR)-based metabolomics is a powerful high-throughput technique that simultaneously detects endogenous compounds representative of a biological state. The low incremental cost and short data collection time of these analyses enable robust experimental design characterized by high reproducibility and ease of sample preparation and measurement proceedings, and also allows for quantitative analysis and in vivo metabolomics studies [14,15,16]. NMR-based metabolomics has been used to express bivalve responses to external disturbance, such as from pathogenic bacteria [17], estrogenic mixtures [18], hepatotoxic microcystins [19], ammonia nitrogen exposure [20], and high ρCO_2_ levels [21]. Proton (^1^H) NMR-based metabolomics has identified osmotic regulation and energy metabolism in *C. hongkongensis* to be disturbed following exposure to Cu, Zn, Pb, and other metals [22,23]. These diverse applications in bivalves suggest that NMR-based metabolomics is a powerful tool for assessing the metabolic response mechanism of stressed bivalves in general, and *C. hongkongensis* in particular.

Differences in the immunological responses of *C. hongkongensis* males and females have seldom been taken into consideration [7,9]. *Vibrio harveyi* is a pathogenic bacterium isolated in our laboratory, which can cause severe vibriosis in *C. hongkongensis*. Lipopolysaccharide (LPS) is a cell wall component of Gram-negative bacteria, and injection of LPS triggers a host immune response [24]. We previously reported gender-related differences in immune responses to LPS and *V. harveyi* in hemocyte of *C. hongkongensis* [25]. Herein we use ^1^H NMR-based metabolomics techniques to examine metabolic changes in hepatopancreas tissues of female and male *C. hongkongensis* following infection with *V. harveyi* and LPS. Our objectives are to identify gender-specific metabolic responses in this oyster following infection and to identify potential new biomarkers for evaluating its health.

## 2. Materials and Methods

### 2.1. Animals and Experimental Design

Healthy post-spawning *C. hongkongensis* (shell length 11.23 ± 0.06 cm) were obtained from a commercial farm in Taishan, Jiangmen, Guangdong Province, China. Oysters were acclimated for 7 days in aerated sand-filtered seawater at 25 °C, salinity 20 ± 1, and pH 8.1 ± 0.1, and regularly fed the algae *Isochrysis galbana* and *Chaetoceros muelleri* at a ration of 2% of tissue per dry twice daily, namely at 08:00 and 16:00. A photoperiod of 12 h light/12 h dark was applied. Low salinity water is prepared by diluting seawater with tap water and used after 2 days of aeration. No mortality occurred during the period of acclimation. After acclimation, oysters were randomly divided into 3 treatments (control, *V. harveyi*, LPS), each with 3 replicates, each replicate containing 20 oysters, in glass aquaria (48 × 28 × 21 cm) with 30 L filtered seawater, in a completely randomized design. 

### 2.2. Challenge Experiment

The bacterium *V. harveyi* was cultivated in liquid 2216E broth at 28 °C for 14 h and centrifuged at 5000× *g* for 10 min. This bacterium was washed twice in sterile seawater before being suspended in sterile seawater at a final concentration of approximately 1 × 107 CFU/mL. LPS (from *Escherichia coli* O111: B4, Sigma-Aldrich, St. Louis, MO, USA) was dissolved in sterile seawater to a concentration of 0.5 mg/mL [25]. Control group oysters were injected with 50 μL sterile aquaculture seawater and the experimental treatment oysters were injected with either 50 μL *V. harveyi* suspension or LPS solution. After 48 h exposure, oyster gender was determined and the oyster hepatopancreas was extracted, snap-frozen in liquid nitrogen, and stored at −80 °C for subsequent metabolite extraction. Gender was determined by light-microscopic examination of mature gonad tissue: large eggs indicated female and motile sperm male.

### 2.3. Metabolite Extraction

Using a water/methanol method, polar metabolites were extracted from hepatopancreas tissues. In brief, 100 mg of hepatopancreas tissue and 1 mL of methanol and water (2:1) were added into a 2 mL centrifuge tube. The mixture was crushed by shaking in a sample freezing grinding machine (Luka, Guangzhou), followed by centrifugation (10 min, 12,000× *g*, 4 °C) and dried in a vacuum centrifugal concentrator. Tissue extracts were resuspended in 600 μL of phosphate buffer in D_2_O, then vortexed and centrifuged (5 min, 3000× *g*, 4 °C). The supernatant (500 μL) was transferred to a 5 mm NMR tube and analyzed by NMR [26]. One-dimensional ^1^H NMR spectra of all samples were obtained at 298K using the 1D NOESYGPPRLD pulse on the Bruker Avance III 600 MHz spectrometer, with 128 scans and a 4 s acquisition time.

### 2.4. Spectral Processing and Statistical Analyses

NMRProcFlow 1.3.10 (INRA UMR 1332 BFP, Bordeaux Metabolomics Facility, Villenave d’Ornon, France) [27] was used to perform PPM calibration, baseline correction, alignment, spectra bucketing, and data normalization of raw ^1^H NMR spectra. The DSS internal standard was taken as the chemical shift reference peak (DSS = 0.0 ppm), and the spectral images within 0.66–10 ppm were integrated to remove the chemical shift region where the water peak was located (4.67–4.86 ppm). PQN (Probabilistic Quotient Normalization) was selected for normalization. NMR spectra were preprocessed using adaptive intelligent bucketing, with buckets with a signal-to-noise ratio > 3 chosen for further investigation. The end result is a 599-bucket matrix. To increase the weight of low-intensity peaks, all NMR spectra were logarithmically transformed before multivariate statistical analysis.

SIMCA 14.1 software (Umetrics, Umeå, Sweden) was used to analyze NMR spectrum datasets. Intrinsic metabolic trends and differential metabolites of *V. harveyi* or LPS exposure were determined using principal component analysis, partial least squares discriminant analysis (PLS-DA), and orthogonal partial least squares discriminant analysis (OPLS-DA). In addition, 200 permutation tests and cross-validation analysis of variance (CV-ANOVA) were used to confirm the significance of the OPLS-DA model. Differential buckets with variable importance in the projection > 1.0 determined by OPLS-DA, and *p*-values < 0.05 determined by two-tailed Student’s *t*-tests were identified. Chenomx NMR Suite 8.6 professional software (Chenomx Inc., Edmonton, AB, Canada) was used to analyze the chemical shift of buckets to complete identification of main metabolites.

### 2.5. Systematic Statistical Metabolic Correlation and Network Analysis

The transformations and sequential chemical reactions of substrates and products involved in diverse catabolic and anabolic processes are described by metabolic pathways [28]. We screened out different metabolites and performed metabolic pathway analysis from KEGG database analysis by MetaboAnalyst 5.0 website [29], and to all the identification of the metabolites of clustering. We used the R software package to identify the KEGG pathways of enriched metabolites in different treatments and to draw correlation heat maps between metabolites and immune-related factors.

## 3. Results

### 3.1. Hepatopancreas ^1^H NMR Spectra

**Figure 1:**
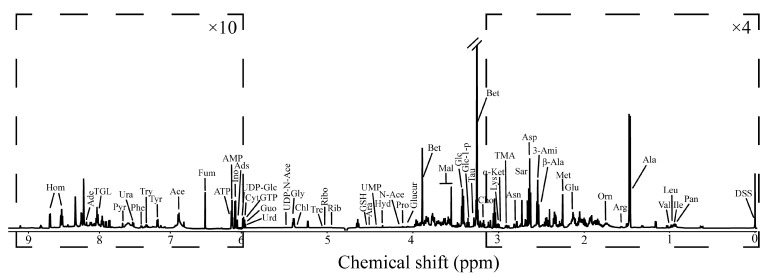
^1^H NMR spectra of *Crassostrea hongkongensis* hepatopancreas extract under infected (control) condition, with spectral regions 0.0–4.8 ppm amplified by a factor of 4, and 4.8–9.9 ppm by 10. Key: Ace, acetate; Ade, adenine; Ads, adenosine; Ala, alanine; AMP, adenosine monophosphate; Ara, arabinose; Arg, arginine; Asn, asparagine; Asp, aspartate; ATP, adenosine triphosphate; Bet, betaine; Chl, chlorogenate; Cho, choline; Cyt, cytosine; DSS, dextran sulfate sodium; Fum, fumarate; Glc, glucose; Glc-1-p, glucose-1-phosphate; Glu, glutamate; Glucur, glucuronate; Gly, glycine; GSH, glutathione; GTP, guanosine triphosphate; Guo, guanosine; Hom, homarine; Hyd, hydroxyacetone; Ile, isoleucine; Ino, inosine; Leu, leucine; Lys, lysine; Mal, malonate; Met, methionine; N-Ace, N-acetylornithine; Orn, ornithine; Pan, pantothenate; Phe, phenylalanine; Pro, proline; Pyr, pyridoxine; Rib, ribose; Ribo, riboflavin; Sar, sarcosine; Tau, taurine; TMA, trimethylamine; TGL, trigonelline; Tre, trehalose; Try, tryptophan; Tyr, tyrosine; UDP-Glc, UDP-glucose; UDP-N-Ace, UDP-N-acetylglucosamine; UMP, uridine monophosphate; Ura, uracil; Urd, uridine; Val, valine; 3-Ami, 3-aminoisobutyrate; α-Ket, α-ketoglutarate acid; β-Ala, β-alanine.

NMR spectra identified 49 different metabolites (Figure 1), including energy metabolism-related metabolites (e.g., glucose, glycogen, ATP, AMP), amino acids (e.g., tryptophan, arginine, phenylalanine, proline, tyrosine) and organic osmolytes (e.g., betaine, taurine, trigonelline). The hepatopancreas ^1^H NMR profile was dominated largely by betaine and taurine, as previously reported for oysters [30,31].

### 3.2. Multivariate Data Analyses

**Figure 2:**
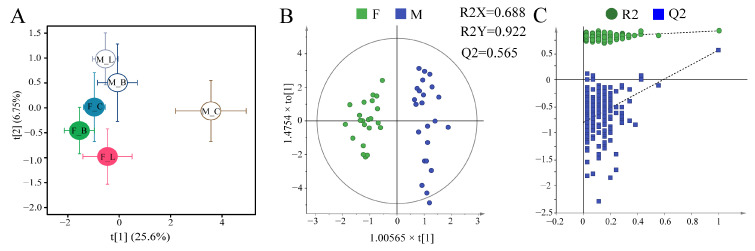
Multivariate statistical analysis plot based on ^1^H NMR spectra of *C. hongkongensis* hepatopancreas tissues. (**A**) PLS-DA plots for all treatments; (**B**) OPLS-DA score plots of male and female oysters (R^2^X = 68.8%, R^2^Y = 0.927, Q^2^ = 0.565, *p* < 0.003); (**C**) permutation test for the model in (**B**). Groups: F, female; M, male; C, control; B, *Vibrio harveyi* infection; L, LPS infection.

To identify metabolic differences between the control and two treatments, and between males and females, PLS-DA analysis was first performed. Treatments were well separated (Figure 2A). To analyze metabolic differences between gender, OPLS-DA was performed on NMR spectral data of males and females from all groups; a clear separation between male and female groups with reliable Q^2^ values, both with *p* values < 0.003 calculated using CV-ANOVA (Figure 2B). We conducted 200 iterations of permutation tests to assess if models were over-fitted to demonstrate that the OPLS-DA model was reliable. Inherent biological and metabolic differences appear to exist between genders.

**Figure 3:**
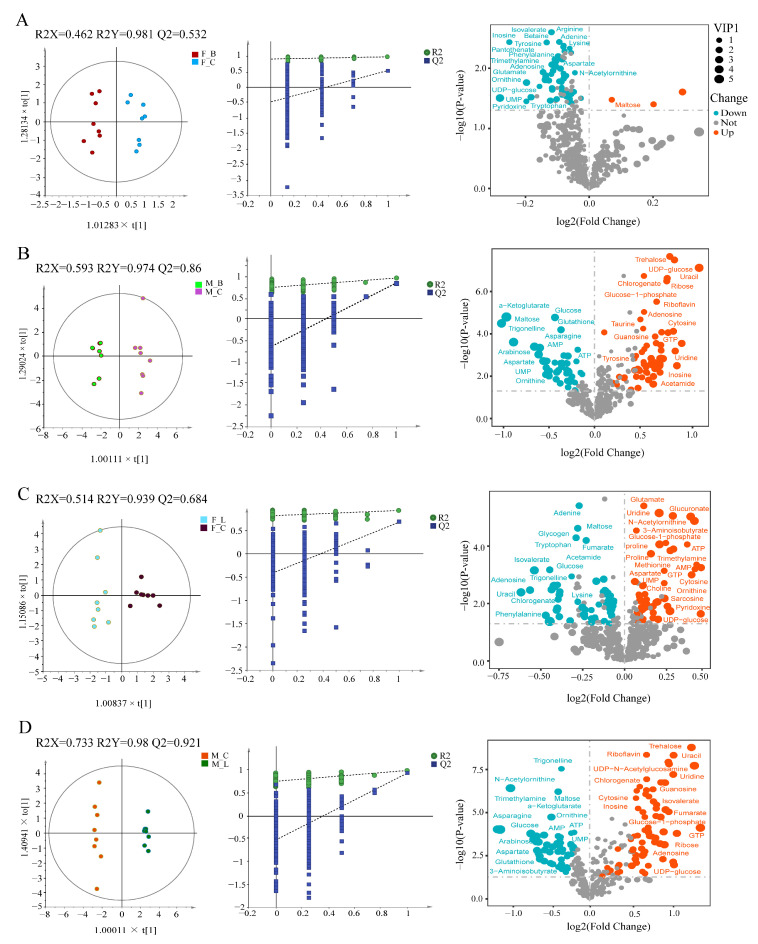
OPLS-DA score plots of ^1^H NMR spectra of *C. hongkongensis* hepatopancreas extract from different paired groups (left panel) and the corresponding model permutation test chart (middle panel) and its corresponding volcano map (right panel). (**A**) F_B vs F_C. (**B**) M_B vs M_C. (**C**) F_L vs F_C. (**D**) M_C vs M_L. Groups: F, female; M, male; C, control; B, *V. harveyi* infection; L, LPS infection.

OPLS-DA score plots (left panels in Figure 3) show good separation between infected treatments and the corresponding control group for males and females. As shown in volcano maps (right panels in Figure 3) and heatmaps (Figure S1), compared with the control group, 19 metabolites were down-regulated and 1 metabolite was up-regulated in *V. harveyi*-infected females. In LPS-infected females, 14 metabolites were down-regulated and 20 metabolites were up-regulated. In *V. harveyi*-infected males, 12 metabolites were down-regulated and 16 metabolites were up-regulated, while in LPS-infected males, 15 metabolites were down-regulated and 16 metabolites were up-regulated. Table S1 provide details of these metabolites.

**Figure 4:**
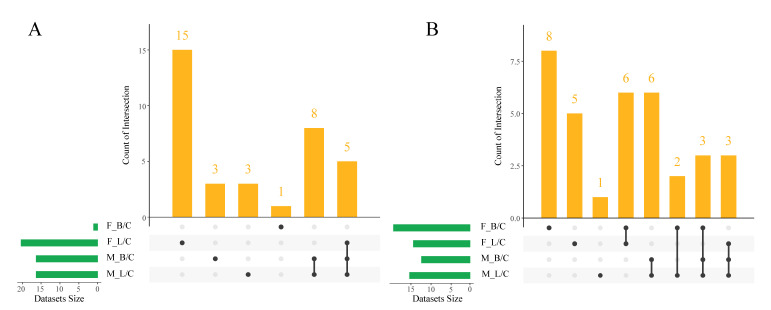
Upset diagram of *C. hongkongensis* hepatopancreas extracts from different paired groups. Increased metabolites (**A**), decreased metabolites (**B**). Groups: F, female; M, male; C, control; B, *V. harveyi* infection; L, LPS infection.

To further analyze changes in differential metabolites in *C. hongkongensis* in different treatments, an Upset diagram was prepared using R language (Figure 4). Compared with control group females, different metabolites increased in female oysters in the two treatments, but the *V. harveyi* treatment had 1 specific increased metabolite, and the LPS group had 15 specific increased metabolites. However, six metabolites were down-regulated in both treatments, while eight metabolites were up-regulated in the *V. harveyi* treatment and five metabolites were down-regulated in the LPS treatment. Compared with the control group male oysters, in the *V. harveyi* treatment eight metabolites were commonly elevated in male oysters, with three particularly elevated, and three were particularly elevated in LPS treatment male oysters. Six metabolites were commonly down-regulated in both treatments, while the *V. harveyi* treatment had no particularly decreased metabolite, and the LPS treatment had one. These metabolites, which are only up- or down-regulated in females or males, suggest that oyster responses to either infection is gender specific. Additionally, different infections in the same gender oysters can lead to different metabolite changes, indicating that oysters are immune-specific for different stresses.

**Figure 5:**
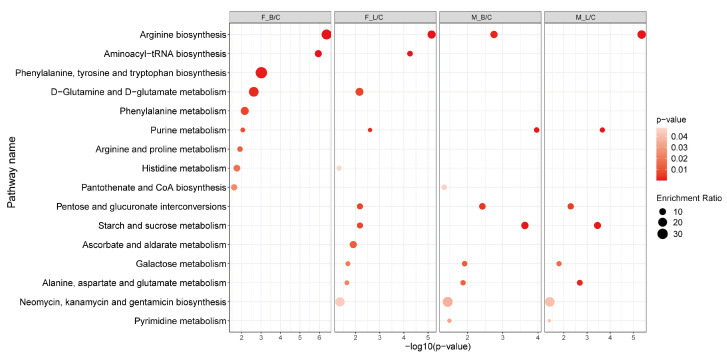
KEGG function analysis based on the differential metabolites from different paired groups. Pathways with *p* values < 0.05 are shown. Groups: F, female; M, male; C, control; B, *V. harveyi* infection; L, LPS infection.

**Figure 6:**
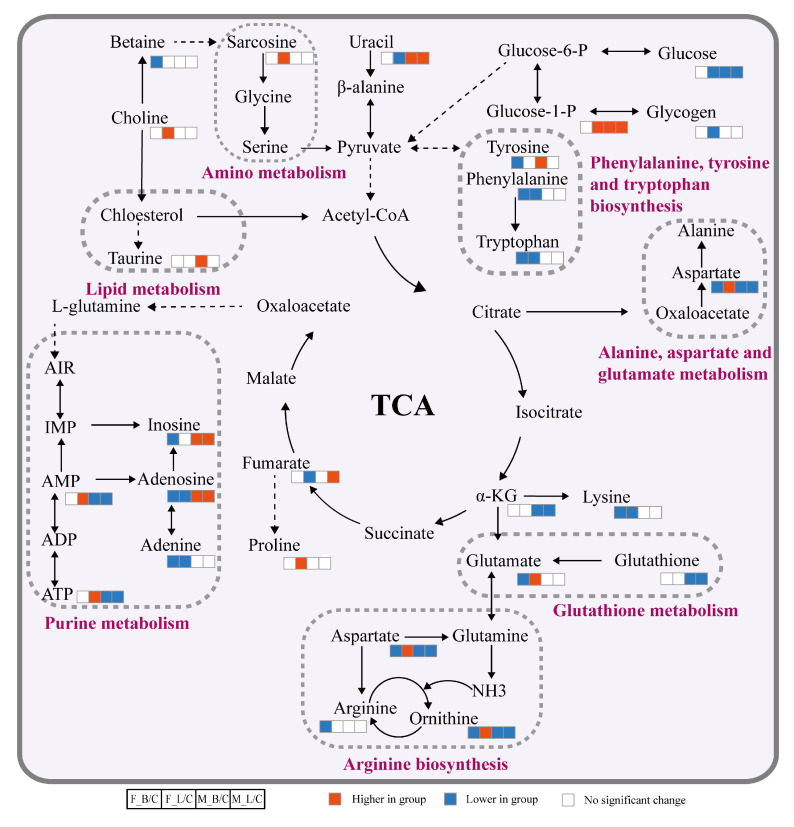
Molecular mechanism of the hepatopancreas response in male and female *C. hongkongensis* to *V. harveyi* and LPS infection according to KEGG. Blue (down-regulated), red (up-regulated), and white (unchanged) boxes reveal changes in levels of differential metabolites compared to controls; ellipses with different background colors indicate different interconnecting pathways. Groups: F, female; M, male; C, control; B, *V. harveyi* infection; L, LPS infection.

KEGG enrichment metabolic pathways prepared by R language clearly differ between male and female oysters (Figure 5). For example, differential metabolites in females occurred mainly in D-glutamine and D-glutamate metabolism, aminoacyl-tRNA biosynthesis and phenylalanine, and tyrosine and tryptophan biosynthesis, while males do not have these enrichment pathways. Additionally, metabolic pathways enriched by either infection in females also differ. For example, differential metabolites in oysters infected with *V. harveyi* were enriched with phenylalanine, tyrosine, and tryptophan biosynthesis and arginine and proline metabolism, while those infected with LPS were enriched with pentose and glucuronate interconversions and galactose metabolism. Based on KEGG pathway analysis, we summarize the different metabolic pathways of oyster infection in hepatopancreas tissues, mainly involving arginine biosynthesis, energy metabolism, glutathione metabolism, and other metabolic pathways (Figure 6).

**Figure 7:**
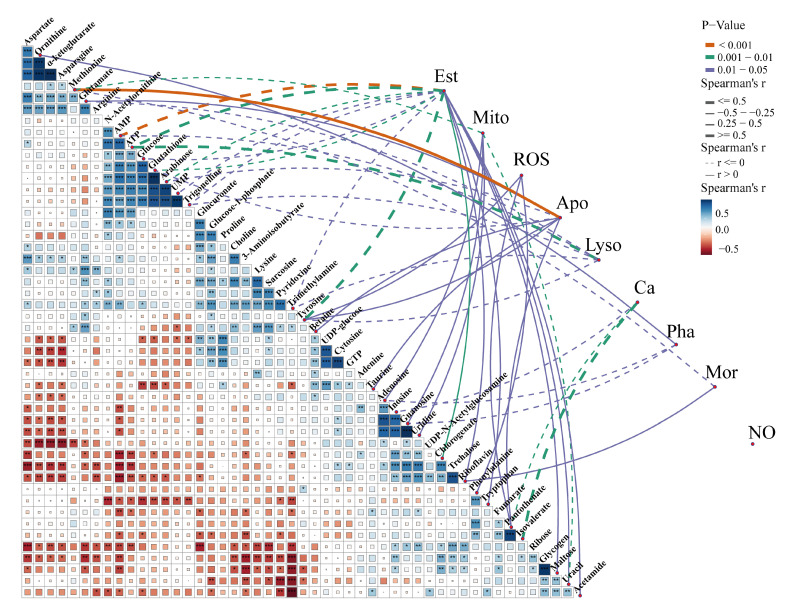
Differential metabolites and immune factors correlated by Spearman’s correlation analysis. A color gradient denoting Spearman’s correlation coefficient is shown for pairwise comparisons of metabolites. Spearman’s correlation coefficients are depicted using line size and line color denotes statistical significance. Lines: dashed, positive correlation; solid, negative connection. Apo, apoptotic ratio; Ca, calcium content; Est, esterase activity; Lyso, lysosome mass; Mito, mitochondrial mass; Mor, mortality; NO, nitric oxide level; Pha, phagocytic ratio; ROS, reactive oxygen species level. *** *p* < 0.001, ** *p* < 0.01, * *p* < 0.05.

A correlation network diagram (Figure 7) was used to represent Spearman’s correlation coefficients between hemocyte immunological parameters and different metabolites. Changes in immunological parameters of granulocytes, including ROS and calcium levels, lysosome and mitochondrial masses, and early apoptotic, NO, phagocytic, and late apoptotic or necrotic ratios of total hemocytes in *C. hongkongensis* during immune stress, have been reported earlier [25]. Significant correlations between parameters may indicate an equilibrium or that immune stimulation in oysters simultaneously modulates parameters. Correlation analysis reveals glucose, AMP, and ATP to be positively correlated, as are proline and choline, and for phenylalanine and tyrosine to be positively correlated with ROS.

## 4. Discussion

Gender-specific differences in physiological response mechanisms are reported for several aquatic invertebrates when exposed to environmental stress. Using NMR-based metabolomics, gender differences have been reported for the mussel *Mytilus edulis* exposed to lower pH, higher temperature and pathogens in seawater [32], and for metabolic responses in gonads of *Perna viridis* to triazophos [33]. We previously reported gender-specific immunological responses in oyster hemocytes following exposure to *V. harveyi* and LPS. Herein, through analysis of OPLS-DA score plots (Figure 2B), we report gender differences in oysters in metabolic responses to two infections.

LPS and *V. harveyi* infection can produce ROS in female oysters [25]. Cell damage occurs when the level of ROS exceeds a cell’s ability to scavenge them, eventually leading to DNA oxidative damage and abnormal protein expression, inducing inflammation and oxidative stress. We herein report proline and choline to be positively correlated, and to significantly increase after LPS infection in females. Antioxidant enzyme activity and antioxidant content are correlated with proline, and by regulating proline metabolism, damage caused by stress can be alleviated [34]. Therefore, we believe that the high expression of proline in females relieves oxidative stress induced by LPS, protecting the oysters, and that the manner of adjustment is gender specific. As components of phospholipids, choline and phosphocholine play important roles in maintaining cell membrane integrity [35]. Therefore, an increase in female oyster hepatopancreas-tissue choline levels following LPS infection indicates that the hepatopancreas membrane is damaged by excessive ROS. In contrast, these metabolites did not change in male oysters after infection, suggesting that male oysters are more capable of coping with *V. harveyi* and LPS stress and maintaining a relatively normal metabolism. Glutathione is an important metabolite that maintains cellular redox balance [36,37] that can be used to enhance antioxidant enzyme activity and antioxidative stress capacity. Glutathione plays a vital role in cellular proliferation, maintaining intracellular redox homeostasis and protecting against oxidative damage [38,39]. In male oysters, glutathione levels decreased with *V. harveyi* and LPS infection, indicating that they mobilized more glutathione to regulate ROS levels after infection, providing further evidence that males are more capable of coping with oxidative stress.

We report phenylalanine and tryptophan levels in female oysters to decrease in both infections, and tyrosine levels following *V. harveyi* infection in female oysters to be down-regulated and in males up-regulated. Oysters are infected, with changes in aromatic amino acids being gender specific. Additionally, we report phenylalanine and tyrosine to be positively correlated with ROS. Because phenylalanine inhibits ROS-induced oxidative damage in gills of grass carp [40], we speculate that decreased phenylalanine reduces the oxidative damage of high ROS levels in female oysters, further supporting the notion that males have a stronger defense mechanism against oxidative stress than females.

In male oysters, metabolites involved in energy metabolism, glucose-1-phosphate, were up-regulated, and glucose, ATP, and AMP were down-regulated in both infections compared with control treatment levels. A decrease in glucose means that male oysters require more energy to resist infection, which will accelerate the hydrolysis of glucose within the body, and generate ATP through the glycolysis pathway and TCA cycle. The process of ATP generation is consistent with this research [41]. Coincidentally, correlation analysis revealed glucose, AMP, and ATP levels to be positively correlated. In female oysters, an increase in ATP, glucose-1-phosphate, and AMP and a decrease in glucose and glycogen occurred following LPS infection. After LPS infection, down-regulation of glucose and glycogen levels promoted the up-regulation of ATP levels. Because the breakdown of glucose and glycogen produces ATP to provide energy to fight infection, but the ATP produced is insufficient to support resistance to LPS infection, the breakdown of glycogen produces glucose-1-phosphate to provide energy. Because none of these metabolites changed after female oysters were infected with *V. harveyi*, females may use different energy metabolism strategies in response to different infections in a manner similar to how the hepatopancreas of the clam *Ruditapes philippinarum* regulates *V. anguillarum* and *V. splendidus* infections using different energy metabolism mechanisms [26]. Collectively, for the same infection conditions, male and female oysters have different energy metabolism mechanisms, indicating that their post-infection energy metabolism mechanism is gender specific. When challenged with *V. harveyi*, male and female *Mytilus galloprovincialis* mussels also induced different energy metabolic responses [42]. For both *V. harveyi* and LPS infections, metabolites involved in energy metabolism such as glucose, ATP, glucose-1-phosphate, and AMP in male oysters changed significantly, while metabolite glucose, ATP, glucose-1-phosphate, AMP, and glycogen in females only changed after LPS infection. This indicates that gender- and immune-specific energy metabolic responses were induced in *C. hongkongensis* and suggests that male oysters have a high antioxidant capacity because most of their energy is used to fight ROS caused by infection.

The TCA cycle is the primary catabolic pathway for energy production. An increase in intermediates in the TCA cycle has been reported in marine bivalves exposed to pathogens [13]. Conversely, the disruption of the TCA cycle shifts energy pathways from aerobic to anaerobic metabolism [43]. For invertebrates, anaerobic metabolism produces high levels of alanine and succinic acid as end products [44]. We found no increase in either succinic acid or alanine in oysters exposed to *V. harveyi* or LPS, suggesting that a shift from aerobic to anaerobic metabolism did not occur. However, fumarate, an intermediate substance of the TCA cycle, decreased in LPS-infected females and increased in *V. harveyi*-infected males, and levels of α-ketoglutarate acid decreased in males in both infections. This indicates that the TCA cycle pathway of oysters in both infections is also gender specific.

Organic osmotic substances (including betaine, trigonelline, and taurine), major components of water-soluble metabolites that often play crucial roles in osmotic regulation of marine mollusks are affected by external influences [45]. Amino acids are involved in marine mollusk osmotic regulation [46]. In male oysters, taurine levels were up-regulated in the *V. harveyi* treatment, while levels of trigonelline and aspartate decreased in both infection treatments. In females, betaine and arginine levels were down-regulated in both infection treatments. In oysters, the depletion of betaine and trigonelline indicates that *V. harveyi* and LPS had disturbed osmotic regulation, as did a decrease in most amino acid levels. Increased taurine may compensate for lost betaine and trigonelline. A similar metabolic response occurred when *V. harveyi* attacked the hepatopancreas of female *M. galloprovincialis* mussels [42]. Accordingly, gender-specific responses to both infections in the oyster hepatopancreas are confirmed by differences in changes in their organic osmotic substances and amino acids.

## 5. Conclusions

The metabolic response of the *C. hongkongensis* hepatopancreas to *V. harveyi* and LPS infection was examined using ^1^H NMR-based metabolomic methods. We conclude that the infection of *V. harveyi* and LPS caused gender- and immune-specific effects on oxidative stress, energy metabolism, the TCA cycle, and in osmotic regulation. The antioxidant capacity of male oysters was stronger than that of females. Overall, in terms of energy metabolism, ATP, AMP, and glucose decreased in males, while ATP and AMP increased in LPS-infected females. In terms of oxidative stress, glutathione decreased in males, and phenylalanine and tryptophan decreased in females, while proline and choline also increased in LPS-infected females. In terms of the TCA cycle, α-ketoglutarate acid decreased in males, and fumarate decreased in LPS-infected females and increased in LPS-infected males. In terms of osmotic regulation, trigonelline decreased in males, taurine increased in *V. harveyi*-infected males, and betaine decreased n *V. harveyi*-infected females. These findings help explain the response mechanisms of different oyster genders to immune stimulation and provide a reference for stress research in marine animals.

## Data Availability

All data underlying this article are available in the main publication and in its Supplementary Material online.

## Supplementary Materials

The following supporting information can be downloaded at: https://www.mdpi.com/article/10.3390/antiox11061178/s1, Figure S1: Heat map analysis of changes in metabolites of hepatopancreas tissues in male and female oysters under *Vibrio harveyi* and LPS infection compared to control groups; Table S1: Summary of metabolic changes of oyster hepatopancreas caused by infection with *Vibrio harveyi* or LPS in different sexes.

## References

[1] Peng J.X., Li Q.Z., Xu L., Wei P.Y., He P.P., Zhang X.Z., Zhang L., Guan J.L., Zhang X.J., Lin Y. (2020). Chromosome-level analysis of the *Crassostrea hongkongensis* genome reveals extensive duplication of immune-related genes in bivalves. Mol. Ecol. Resour..

[2] Zhang Y.H., Li J., Qin Y.P., Zhou Y.E., Zhang Y., Yu Z.N. (2017). A comparative study of the survival, growth and gonad development of the diploid and triploid Hong Kong oyster, *Crassostrea hongkongensis* (Lam & Morton 2003). Aquac. Res..

[3] Qin Y.P., Li X.Y., Liao Q.L., Li J., Ma H.T., Mo R.G., Zhang Y.H., Yu Z.N. (2021). Comparative study on the growth, survival, gonad development and trait segregation of F2 hybrids and their grandparent species (*Crassostrea ariakensis* and *C. hongkongensis*). Aquaculture.

[4] Yang Y., Qin Y.P., Zhang A.J., Zhou Y.Y., Li J., Liao Q.L., Shi G.P., Yu Z.N., Pan Y., Zhang Y.H. (2022). Cloning and characterization of a novel hydrolase gene from Hong Kong oyster *Crassostrea hongkongensis*. Aquacult. Rep..

[5] Zhou Y.Y., Liu K.N., Li X.Y., Qin Y.P., Zhang Y.H., Zhang Y., Xiang Z.M., Ma H.T., Li J., Yu Z.N. (2020). Molluscan Beclin-1 is involved in the innate immune response by regulating the autophagosomes formation in *Crassostrea hongkongensis*. Aquacult. Rep..

[6] Fu D.K., Zhang Y., Yu Z.N. (2011). Cloning and expression analysis of a ubiquitin gene (*Ub_L40_*) in the haemocytes of *Crassostrea hongkongensis* under bacterial challenge. Chin. J. Oceanol. Limnol..

[7] Wang F.X., Xiao S., Zhang Y., Zhang Y.H., Liu Y., Yan Y., Xiang Z.M., Yu Z.N. (2015). *ChAkt1* involvement in orchestrating the immune and heat shock responses in *Crassostrea hongkongensis*: Molecular cloning and functional characterization. Fish Shellfish Immunol..

[8] Xiang Z.M., Qu F.F., Qi L., Ying T., Li J., Shu X., Yu Z.N. (2014). Cloning and characterization of an apoptosis-related DNA fragmentation factor (DFF) from oyster, *Crassostrea hongkongensis*. Fish Shellfish Immunol..

[9] Qu F.F., Xiang Z.M., Zhang Y., Li J., Xiao S., Zhang Y.H., Mao F., Ma H.T., Yu Z.N. (2016). A novel p38 MAPK indentified from *Crassostrea hongkongensis* and its involvement in host response to immune challenges. Mol. Immunol..

[10] Tong Y., Zhang Y., Huang J.M., Xiao S., Zhang Y.H., Li J., Chen J.H., Yu Z.N. (2015). Transcriptomics analysis of *Crassostrea hongkongensis* for the discovery of reproduction-related genes. PLoS ONE.

[11] Cao C., Wang W.X. (2017). Chronic effects of copper in oysters *Crassostrea hongkonggensis* different exposure regimes as shown by NMR-based metabolomics. Environ. Toxicol. Chem..

[12] Luo L.Z., Ke C.H., Guo X.Y., Shi B., Huang M.Q. (2014). Metal accumulation and differentially expressed proteins in gill of oyster (*Crassostrea hongkongensis*) exposed to long-term heavy metal-contaminated estuary. Fish Shellfish Immunol..

[13] Nguyen T.V., Alfaro A.C. (2020). Metabolomics investigation of summer mortality in New Zealand Greenshell (TM) mussels (*Perna canaliculus*). Fish Shellfish Immunol..

[14] Cappello T., Giannetto A., Parrino V., Marco G.D., Mauceri A., Maisano M. (2018). Food safety using NMR-based metabolomics: Assessment of the Atlantic bluefin tuna, *Thunnus thynnus*, from the Mediterranean Sea. Food Chem. Toxicol..

[15] Cappello T., Marco G.D., Conti G.O., Giannetto A., Ferrante M., Mauceri A., Maisano M. (2021). Time-dependent metabolic disorders induced by short-term exposure to polystyrene microplastics in the Mediterranean mussel *Mytilus galloprovincialis*. Ecotoxicol. Environ. Saf..

[16] Zitouni N., Cappello T., Missawi O., Boughattas I., Marco G.D., Belbekhouche S., Mokni M., Alphonse V., Guerbej H., Bousserrhine N. (2022). Metabolomic disorders unveil hepatotoxicity of environmental microplastics in wild fish *Serranus scriba* (Linnaeus 1758). Sci. Total Environ..

[17] Frizzo R., Bortoletto E., Riello T., Leanza L., Schievano E., Venier P., Mammi S. (2021). NMR metabolite profiles of the bivalve mollusc *Mytilus galloprovincialis* before and after immune stimulation with *Vibrio splendidus*. Front. Mol. Biosci..

[18] Islam R., Melvin S.D., Yu R.M.K., O’Connor W.A., Tran T.K.A., Andrew-Priestley M., Leusch F.D.L., MacFarlane G.R. (2021). Exposure to estrogenic mixtures results in tissue-specific alterations to the metabolome of oysters. Aquat. Toxicol..

[19] Yang W., Ye Y.F., Lu K.H., Zheng Z.M., Zhu J.Y. (2022). NMR-based metabolomic responses of freshwater gastropod *Bellamya aeruginosa* to MC-producing and non MCproducing *Microcystis aeruginosa*. J. Oceanol. Limnol..

[20] Lu J., Yao T., Shi S.K., Ye L.T. (2022). Effects of acute ammonia nitrogen exposure on metabolic and immunological responses in the Hong Kong oyster *Crassostrea hongkongensis*. Ecotoxicol. Environ. Saf..

[21] Wei L., Wang Q., Wu H.F., Ji C.L., Zhao J.M. (2015). Proteomic and metabolomic responses of Pacific oyster *Crassostrea gigas* to elevated ρCO2 exposure. J. Proteom..

[22] Cao C., Wang W.X. (2016). Bioaccumulation and metabolomics responses in oysters *Crassostrea hongkongensis* impacted by different levels of metal pollution. Environ. Pollut..

[23] Ji C.L., Wang Q., Wu H.F., Tan Q.G., Wang W.X. (2015). A metabolomic investigation of the effects of metal pollution in oysters *Crassostrea hongkongensis*. Mar. Pollut. Bull..

[24] Rungrassamee W., Maibunkaew S., Karoonuthaisiri N., Jiravanichpaisal P. (2013). Application of bacterial lipopolysaccharide to improve survival of the black tiger shrimp after *Vibrio harveyi* exposure. Dev. Comp. Immunol..

[25] Lu J., Shi Y.Y., Yao T., Bai C.M., Jiang J.Z., Ye L.T. (2021). Gender Differences in Hemocyte Immune Parameters of Hong Kong Oyster *Crassostrea hongkongensis* During Immune Stress. Front. Immunol..

[26] Liu X.L., Ji C.L., Zhao J.M., Wu H.F. (2013). Differential metabolic responses of clam *Ruditapes philippinarum* to *Vibrio anguillarum* and *Vibrio splendidus* challenges. Fish Shellfish Immunol..

[27] Jacob D., Deborde C., Lefebvre M., Maucourt M., Moing A. (2017). NMRProcFlow: A graphical and interactive tool dedicated to 1D spectra processing for NMR-based metabolomics. Metabolomics.

[28] Elliott P., Posma J.M., Chan Q., Garcia-Perez I., Wijeyesekera A., Bictash M., Ebbels T.M.D., Ueshima H., Zhao L.C., van Horn L. (2015). Urinary metabolic signatures of human adiposity. Sci. Transl. Med..

[29] Pang Z.Q., Chong J., Zhou G.Y., Morais D.A.d.L., Chang L., Barrette M., Gauthier C., Jacques P.-E., Li S.Z., Xia J.G. (2021). MetaboAnalyst 5.0: Narrowing the gap between raw spectra and functional insights. Nucleic Acids Res..

[30] Sokolov E.P., Sokolova I.M. (2019). Compatible osmolytes modulate mitochondrial function in a marine osmoconformer *Crassostrea gigas* (Thunberg, 1793). Mitochondrion.

[31] Eymann C., Gotze S., Bock C., Guderley H., Knoll A.H., Lannig G., Sokolova I.M., Aberhan M., Portner H.O. (2020). Thermal performance of the European flat oyster, *Ostrea edulis* (Linnaeus, 1758)-explaining ecological findings under climate change. Mar. Biol..

[32] Ellis R.P., Spicer J.I., Byrne J.J., Sommer U., Viant M.R., White D.A., Widdicombe S. (2014). ^1^H NMR Metabolomics Reveals Contrasting Response by Male and Female Mussels Exposed to Reduced Seawater pH, Increased Temperature, and a Pathogen. Environ. Sci. Technol..

[33] Zhang L.B., Sun W., Zhang Z., Chen H.G., Jia X.P., Cai W.G. (2017). Gender-specific metabolic responses in gonad of mussel *Perna viridis* to triazophos. Mar. Pollut. Bull..

[34] Zhang D.J., He S., Ming T.H., Lu C.Y., Zhou J., Su X.R. (2017). A metabonomic analysis on the response of *Enterobacter cloacae* from coastal outfall for land-based pollutant under phoxim stress. Arch. Microbiol..

[35] Li M.H., Wang J.S., Lu Z.G., Wei D.D., Yang M.H., Kong L.Y. (2014). NMR-based metabolomics approach to study the toxicity of lambda-cyhalothrin to goldfish (*Carassius auratus*). Aquat. Toxicol..

[36] Yang C.Y., Hao R.J., Du X.D., Wang Q.H., Deng Y.W., Sun R.J. (2019). Response to different dietary carbohydrate and protein levels of pearl oysters (*Pinctada fucata martensii*) as revealed by GC-TOF/MS-based metabolomics. Sci. Total Environ..

[37] Tweeddale H., Notley-Mcrobb L., Ferenci T. (1998). Effect of Slow Growth on Metabolism of *Escherichia coli*, as Revealed by Global Metabolite Pool (“Metabolome”) Analysis. J. Bacteriol..

[38] Liu J.D., Liu W.B., Zhang D.D., Xu C.Y., Zhang C.Y., Zheng X.C., Chi C. (2020). Dietary reduced glutathione supplementation can improve growth, antioxidant capacity, and immunity on Chinese mitten crab, *Eriocheir sinensis*. Fish Shellfish Immunol..

[39] Xia Z.Q., Wu S.J. (2018). Effects of glutathione on the survival, growth performance and non-specific immunity of white shrimps (*Litopenaeus vannamei*). Fish Shellfish Immunol..

[40] Feng L., Li W., Liu Y., Jiang W.D., Kuang S.Y., Wu P., Jiang J., Tang L., Tang W.N., Zhang Y.G. (2017). Protective role of phenylalanine on the ROS-induced oxidative damage, apoptosis and tight junction damage via Nrf2, TOR and NF-kB signalling molecules in the gill of fish. Fish Shellfish Immunol..

[41] Rui L.Y. (2014). Energy Metabolism in the Liver. Compr. Physiol..

[42] Liu X.L., Sun H.S., Wang Y.Y., Ma M.W., Zhang Y.M. (2014). Gender-specific metabolic responses in hepatopancreas of mussel *Mytilus galloprovincialis* challenged by *Vibrio harveyi*. Fish Shellfish Immunol..

[43] Nguyen T.V., Alfaro A., Arroyo B.B., Leon J.A.R., Sonnenholzner S. (2021). Metabolic responses of penaeid shrimp to acute hepatopancreatic necrosis disease caused by *Vibrio parahaemolyticus*. Aquaculture.

[44] Sun X.J., Tu K., Li L., Biao W., Wu L., Liu Z.H., Zhou L.Q., Tian J.T., Yang A.G. (2021). Integrated transcriptome and metabolome analysis reveals molecular responses of the clams to acute hypoxia. Mar. Environ. Res..

[45] Lu J., Shi Y.Y., Wang S.H., Chen H., Cai S.H., Feng J.H. (2016). NMR-based metabolomic analysis of *Haliotis diversicolor* exposed to thermal and hypoxic stresses. Sci. Total Environ..

[46] Li F., Meng X.J., Wang X.Q., Ji C.L., Wu H.F. (2021). Graphene-triphenyl phosphate (TPP) co-exposure in the marine environment: Interference with metabolism and immune regulation in mussel *Mytilus galloprovincialis*. Ecotoxicol. Environ. Saf..

