# Supplementary material for: Gender-Specific Metabolic Responses of Crassostrea hongkongensis to Infection with Vibrio harveyi and Lipopolysaccharide
Source: Antioxidants (Basel). 2022 Jun 15;11(6):1178. doi: 10.3390/antiox11061178 (PMC9220117; doi:10.3390/antiox11061178)
Supplement: Supplementary file 1 [file antioxidants-11-01178-s001.zip › antioxidants-1743320-supplementary.pdf]

Gender-specific metabolic responses of *Crassostrea hongkongensis* to infection with *Vibrio harveyi* and lipopolysaccharide

Lijuan Ma [a, b], Jie Lu [a *], Tuo Yao [a], Lingtong Ye [c], Jiangyong Wang [d *]

[a] *Key Laboratory of South China Sea Fishery Resources Exploitation & Utilization, Ministry of Agriculture, South China Sea Fisheries Research Institute, Chinese Academy of Fishery Sciences, Guangzhou 510300, China*

[b] *Chinese Academy of Agricultural Sciences, Beijing 100081, China*

[c] *Key Laboratory of Aquatic Product Processing, Ministry of Agriculture and Rural Affairs, South China Sea Fisheries Research Institute, Chinese Academy of Fishery Sciences, Guangzhou 510300, China*

[d] *Huizhou University, Huizhou 516007, China*

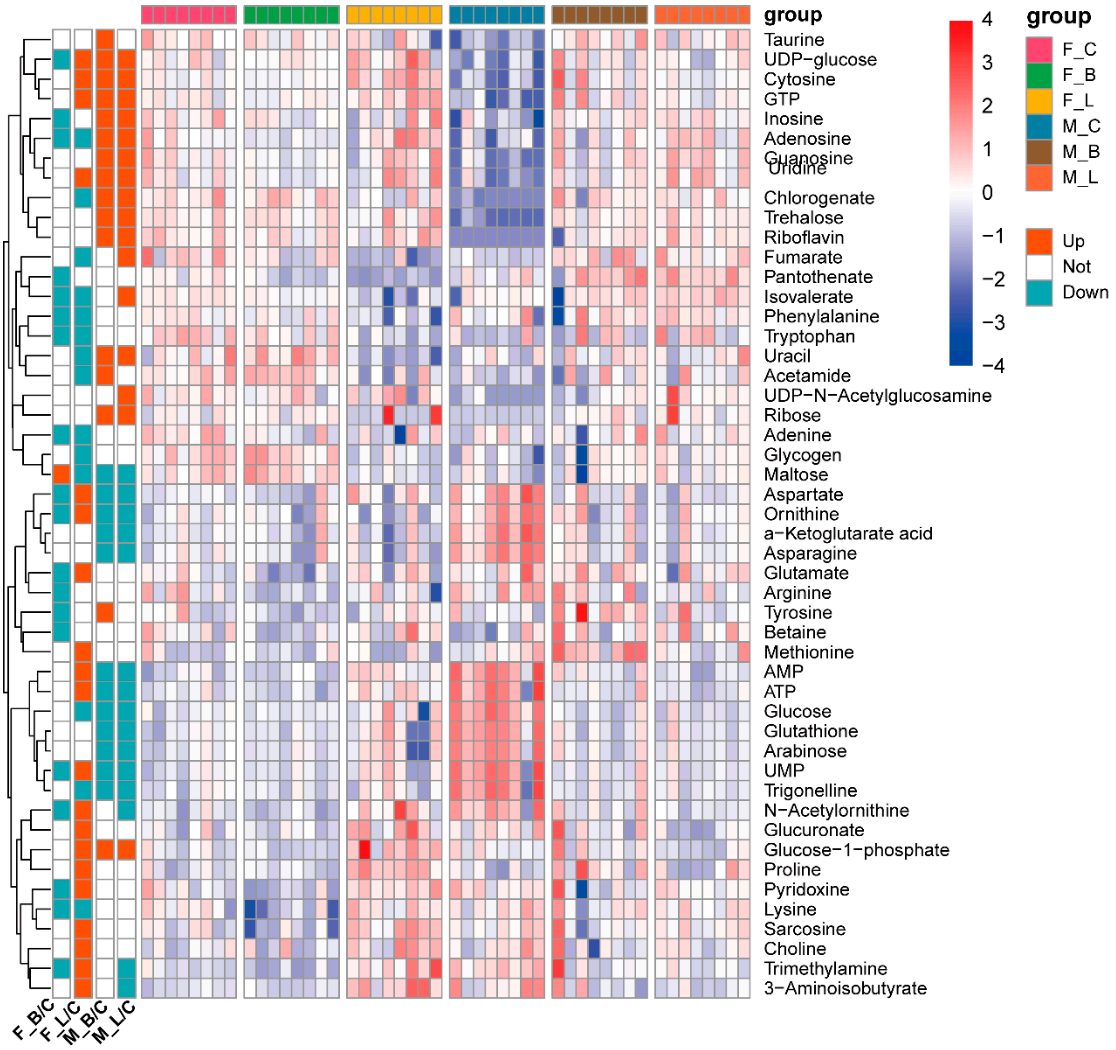

Figure S1. Heat map analysis of changes in metabolites of hepatopancreas tissues in male and female oysters under *Vibrio harveyi* and LPS infection compared to control groups. Groups: F, female; M, male; C, control; B, *V. harveyi* infection; L, LPS infection.

Table S1 Summary of metabolic changes of oyster hepatopancreas caused by infection with *Vibrio harveyi* or LPS in different sexes.

Metabolites	F_B/C			F_L/C			M_B/C			M_L/C		
	FC [a]	VIP [b]	*p*-value [c]	FC	VIP	*p*-value	FC	VIP	*p*-value	FC	VIP	*p*-value
Adenine	0.96	1.41	0.038	0.88	1.43	0.037						
Pyridoxine	0.82	4.04	0.031	1.14	2.03	0.035						
Uracil				0.65	2.82	0.004	1.75	2.07	<0.001	1.59	1.49	<0.001
Phenylalanine	0.92	11.26	0.012	0.75	1.92	0.017						
Tryptophan	0.87	1.38	0.035	0.73	1.56	< 0.001						
Tyrosine	0.92	1.68	0.021				1.15	1.02	0.019			
Acetamide				0.72	2.08	0.025	1.20	1.45	0.010			
Fumarate				0.75	1.34	0.002				1.16	1.09	0.026
Inosine	0.92	1.83	0.007				1.25	1.56	0.011	1.26	1.43	0.009
Adenosine	0.88	2.45	0.030	0.67	2.18	0.003	1.31	1.54	0.043	1.43	1.98	0.004
UDP-glucose	0.89	1.13	0.020	1.36	2.14	0.025	1.58	2.64	0.002	1.44	1.72	0.007
Cytosine				1.22	2.19	0.002	1.64	2.53	0.003	1.44	1.47	0.002
GTP				1.23	1.83	0.024	1.53	2.29	0.001	1.41	1.47	0.001
Guanosine							1.45	1.60	0.002	1.59	1.84	<0.001
Uridine				1.09	1.23	0.010	1.48	1.74	0.003	1.67	2.14	<0.001
UDP-N-Acetylglucosamine										1.51	1.39	<0.001
Glycogen				0.88	2.10	0.001						
Maltose	1.05	1.53	0.033	0.91	1.86	0.001	0.81	2.5	0.001	0.86	1.70	<0.001
Chlorogenate				0.83	1.96	0.013	1.82	2.21	<0.001	1.79	2.70	<0.001
Trehalose							2.21	3.86	<0.001	2.29	3.77	<0.001
Riboflavin							1.94	2.55	<0.001	2.23	3.06	<0.001
Ribose							1.65	1.83	0.001	1.95	2.37	0.009
Glucose				0.91	1.74	0.003	0.74	2.88	<0.001	0.73	2.63	<0.001
Glutathione							0.63	4.12	<0.001	0.62	3.74	<0.001
Arabinose							0.67	3.56	0.001	0.65	3.42	<0.001
AMP				1.17	2.17	0.006	0.77	2.09	0.003	0.69	2.65	<0.001
UMP	0.92	1.91	0.002	1.08	1.32	0.002	0.69	2.40	0.008	0.67	2.32	0.004
Trigonelline				0.84	1.47	0.028	0.81	1.76	0.016	0.80	1.72	0.008
ATP				1.08	1.03	0.034	0.83	1.41	0.018	0.84	1.69	0.002
N-Acetylornithine	0.94	1.76	0.013	1.12	1.48	0.031				0.85	1.12	<0.001
Glucuronate				1.06	1.34	0.044						
Glucose-1-phosphate				1.11	2.09	0.013	1.56	2.30	0.010	1.80	3.07	<0.001
Proline				1.08	1.40	<0.001						
Taurine							1.07	1.27	<0.001			
Betaine	0.98	1.10	0.031									

Choline				1.05	1.23	0.022						
Ornithine	0.92	2.34	0.009	1.12	1.51	0.012	0.83	1.47	0.017	0.83	1.28	0.003
Lysine	0.92	2.54	0.008	0.88	1.27	0.030						
α-Ketoglutarate							0.87	1.24	0.002	0.86	1.12	<0.001
Asparagine							0.79	1.81	0.001	0.77	1.66	<0.001
Trimethylamine	0.91	2.14	0.034	1.19	2.60	0.003				0.864	1.15	<0.001
Aspartate	0.95	1.41	0.032	1.06	1.17	0.020	0.82	1.10	0.006	0.810	1.06	0.001
Sarcosine				1.07	1.45	0.007						
3-Aminoisobutyrate				1.31	2.65	0.001				0.83	1.25	0.016
Glutamate	0.95	1.32	0.036	1.06	1.05	0.004						
Methionine				1.16	3.03	0.001						
Arginine	0.90	2.04	0.013									
Pantothenate	0.95	1.00	0.004									
Isovalerate	0.93	1.69	0.003	0.74	3.53	0.002				1.17	1.27	0.020

F, female group; M, male group; C, control group; B, *Vibrio harveyi* infection group; L, LPS infection group. [a] fold change. [b] variable importance in the projection. [c] *p*-value, representing the significance of the metabolite changes.
